# Neuroprotection for Ischemic Stroke: Moving Past Shortcomings and Identifying Promising Directions

**DOI:** 10.3390/ijms14011890

**Published:** 2013-01-17

**Authors:** Ryan C. Turner, Brandon Lucke-Wold, Noelle Lucke-Wold, Alisa S. Elliott, Aric F. Logsdon, Charles L. Rosen, Jason D. Huber

**Affiliations:** 1 Department of Neurosurgery, One Medical Center Drive, West Virginia University School of Medicine, P.O. Box 9183, Morgantown, WV 26506, USA; E-Mails: rcturner@hsc.wvu.edu (R.C.T.); bwold@mix.wvu.edu (B.L.-W.); aselliott@hsc.wvu.edu (A.S.E.); 2 The Center for Neuroscience, West Virginia University School of Medicine, Morgantown, WV 26506, USA; E-Mails: amoutrie@mix.wvu.edu (N.L.-W.); logsdoa@gmail.com (A.F.L.); jdhuber@hsc.wvu.edu (J.D.H.); 3 Department of Health Restoration, West Virginia University School of Nursing, Morgantown, WV 26506, USA; 4 Department of Basic Pharmaceutical Sciences, West Virginia University School of Pharmacy, Morgantown, WV 26506, USA

**Keywords:** ischemic stroke, comorbidity, aging, macrophage polarization

## Abstract

The translation of neuroprotective agents for ischemic stroke from bench-to-bedside has largely failed to produce improved treatments since the development of tissue plasminogen activator (tPA). One possible reason for lack of translation is the failure to acknowledge the greatest risk factor for stroke, age, and other common comorbidities such as hypertension, obesity, and diabetes that are associated with stroke. In this review, we highlight both mechanisms of studying these factors and results of those that have been addressed. We also discuss the potential role of other lifestyle factors associated with an increased stroke risk such as sleep fragmentation and/or deprivation. Furthermore, many proposed therapeutic agents have targeted molecular mechanisms occurring soon after the onset of ischemia despite data indicating delayed patient presentation following ischemic stroke. Modulating inflammation has been identified as a promising therapeutic avenue consistent with preliminary success of ongoing clinical trials for anti-inflammatory compounds such as minocycline. We review the role of inflammation in stroke and in particular, the role of inflammatory cell recruitment and macrophage phenotype in the inflammatory process. Emerging evidence indicates an increasing role of neuro-immune crosstalk, which has led to increased interest in identification of peripheral biomarkers indicative of neural injury. It is our hope that identification and investigation of factors influencing stroke pathophysiology may lead to improved therapeutics.

## 1. Introduction

### 1.1. Concept of Neuroprotection in Ischemic Stroke

Following the onset of ischemic stroke, cerebral blood flow is disrupted throughout the affected region of the brain. Blood flow disruption to the tissue is not uniform due to the presence of collateral circulation resulting in a flow gradient. Consequently, a “core” region of infarct develops in which flow is severely reduced, often approximating upwards of a 90% decrement, and tissue undergoes necrosis within minutes as insufficient adenosine triphosphate (ATP) is present to maintain homeostatic ionic gradients and metabolic functions [1]. Surrounding this core region in which blood flow is severely reduced is the “penumbra”, a tissue distribution in which flow is less severely reduced and remains typically around 35% of baseline flow [1]. Characterized by normal cellular membrane potential but a disruption in the ability for normal action potential firing, tissue initially residing within the penumbral region progresses to cellular death, ultimately resulting in an expanded core lesion without restoration of cerebral blood flow [2]. As such, therapeutic development has focused on achieving reperfusion via tissue plasminogen activator (tPA) and/or restoring homeostasis via administration of ‘neuroprotective’ pharmacologic agents intended to disrupt the ischemic injury cascade.

### 1.2. Current Status of Neuroprotectant Development: Drug Development Shortcomings

Investigation of neuroprotection and the associated concepts have gone through various periods of apparent focus as described by Lo previously [2]. Early studies, initiated by Astrup and colleagues, focused on understanding the effect of ischemia produced across a range of cerebral blood flow measurements on neurophysiology and electrical conduction [1]. The second phase of investigation sought to identify molecular mechanisms of injury with an emphasis on the acute period of injury. Following molecular biology studies was the discovery and implementation of numerous imaging modalities in both preclinical and clinical studies for assessing both changes in blood flow and the evolution of both the penumbra and infarct core [2]. While these various developments have provided much insight and utility in both preclinical and clinical studies, successful translation of a neuroprotectant from bench-to-bedside has yet to occur despite identification of over 1000 “successful” neuroprotectants in preclinical studies and initiation of over 100 clinical trials [3].

The reasons for lack of translation and failure are presently unknown but clear discrepancies exist between preclinical models and the clinical population most commonly afflicted by ischemic stroke. These include the age and general health of the population being studied as well as the time point of treatment initiation. Similarly, the endpoints frequently used in preclinical and clinical studies are often different. Furthermore, the vast majority of preclinical work has been conducted in rodents, species that are more distant phylogenetically from the human population in comparison to nonhuman primates [4]. Nonhuman primates more closely resemble humans in vascular anatomy, experience striatal damage following ischemic stroke more comparably to the human, and are gyrencephalic in nature like humans [4]. Consequently, nonhuman primates represent perhaps the most ideal preclinical model but are frequently not utilized due to cost, lack of availability, and expertise required for care and use. For these reasons, nonhuman primate models are not discussed further.

The following sections elaborate on these concepts in an attempt to convey the contribution of common comorbidities seen in the clinical population to stroke risk and outcome. Furthermore, the potential impact of other lifestyle factors such as sleep fragmentation and/or deprivation, often related to an altered sleep-wake cycle by something such as shift work, is discussed.

## 2. Improving Animal Models: Role of Comorbidities & Lifestyle

### 2.1. Accounting for the Aging Process: Effects on Inflammation

Age is the greatest risk factor for ischemic stroke yet has frequently not been emphasized in preclinical studies. Aging shifts the body to a pro-inflammatory state [5,6] leading to greater susceptibility to infection and injury as well as eventual immune exhaustion [7]. Aging also increases oxidative stress and immunosenescence [8]. Following stroke, an aged brain suffers greater blood brain barrier (BBB) disruption than a young brain [9]. The effects of aging are mediated through changes in astrocytes, macrophages, and microglia.

#### 2.1.1. Astrocytes

Astrocytes have specific functions following stroke, which make them an excellent target for investigating therapeutic pathways. Astrocytes typically buffer potassium channels and maintain the BBB [10]. Following injury, the genes Lcn2 and Serpina3n, genes associated with astrogliosis, are induced. Activated astrocytes provide protection for an ischemic brain [11]. As the brain ages, astrocytes are subjected to a form of neurosenescence. Particular biomarkers related to astrocytes are increased such as glial fibrillary acidic protein (GFAP), pro-inflammatory cytokines, and protein aggregates thought to induce cellular death [12]. These age-specific changes cause reduced astrocyte-mediated neuroprotection following stroke [13]. Although further investigation is warranted, it is important and necessary to use aged animal models in order to better understand pathway alterations triggered by senescence.

#### 2.1.2. Macrophages

Macrophages have a dichotomous role in stroke injury. M1-type macrophages are involved with inflammatory tissue damage whereas M2-type macrophages are immunoregulators and help with wound healing [14]. Macrophages accumulate following stroke but decrease in number if tissue plasminogen activator (tPA) is given within the suggested time window [15]. It is currently unknown if this macrophage decrease is specific for the M1 or M2-type. Macrophage production of interleukin-6 (IL-6) and reactive oxygen species (ROS) increases with age [16]. Popa-Wagner’s group proposed that the phagocytic activity of brain macrophages drastically increases following infarct in aged animals. This increase results in production of free radicals and increased infarct volume [17]. A possible mechanism for the increased infarct volume is the enhanced expression of CPP32, PARP, and TNF-α [18]. In light of these findings, it will be important in future studies to isolate specific inflammatory pathways in aged-animal models.

#### 2.1.3. Microglia

Microglia undergo a transition from a neuroprotective role in young brains to a neurotoxic role in aged brains. It is thought that this transition involves changes in iron accumulation [19]. Increased iron uptake causes decreased viability of microglia and the release of pro-apoptotic factors [20]. Furthermore, aged microglia secrete excess IL-6 and TNF-α. These cytokines activate neurodegenerative pathways [21]. In stark contrast, young-adult microglia augment hippocampal neurogenesis [22]. Another difference between young and old subjects is the presence of age-altered surface markers such as major histocompatibility complex II (MHCII) and anti-CD68 (ED-1) [23]. Since profound changes in microglia occur with age, it will be necessary to examine stroke injury using aged animal models.

#### 2.1.4. Challenges Associated with Aged Models of Ischemic Stroke

The acquisition and use of aged animals is notably more expensive and difficult than the use of young-adult animals more commonly seen in the literature. Acquiring aged animals often requires the establishment of an aging colony either at the research institution or animal supplier, an expensive endeavor due to *per diem* charges associated with animal care. This often results in aged animals being on the order of ten times more expensive than the young-adult animals frequently used based on the experience of the authors’. The cost is further heightened by mortality experienced during the aging process or transport, approximately 10% by the 18–20 month age in our experience.

In addition to cost of acquisition and use, various technical and methodological challenges are associated with the use of aged rodents. As ischemic stroke is most commonly produced via intraluminal suture occlusion or the insertion of an embolus, uniform vascular anatomy across experimental groups is clearly desirable. This is often not the case when utilizing aged animals as an increased variance in size, with the animals ranging from 300 to 550 grams, is often seen with aging. As such, size and/or placement of the suture/embolus may need to be altered depending on the model of ischemia utilized [24]. Furthermore, anesthesia is potentially complicated by subclinical underlying respiratory or cardiovascular disease, factors that may lead to death prior to experimental completion. Perhaps most importantly, the mortality rate associated with use of aged animals is frequently higher when compared to young-adult animals [25].

### 2.2. Effect of Comorbidities on Stroke Risk & Outcome

Comorbidities have long been known to increase risk for myocardial infarction and stroke. Until recently, individual diseases such as diabetes and hypertension were studied in isolation. Clinicians and researchers alike are now focusing on how multiple variables interact, and subsequently how they increase the threat for vascular disorder [26]. The metabolic syndrome is strongly associated with increased hazard risk for ischemic stroke [27]. It consists of hypertension, insulin resistance, obesity, and hypertriglyceridemia [28]. Although the causes of this syndrome are still under investigation, it is known that sedentary lifestyle, poor diet, and genetics play a role [29]. The following sections will address the current pre-clinical models for diabetes, hypertension, and obesity.

#### 2.2.1. Diabetic Models

In Type II diabetes mellitus, a key factor of disease pathology is hyperinsulinemia. Hyperinsulinemia causes an elevation of advanced glycosylated end-products (AGEs), and AGEs lead to overproduction of reactive oxygen species (ROS). ROS ultimately cause tissue damage and vascular disruption [30]. It is therefore necessary to use models where insulin resistance and insulin levels can be experimentally manipulated prior to stroke.

One such model is the high-fat, streptozotocin-treated Type II diabetic rat [31]. The Ye group used this model to study the effects of diabetes on subsequent stroke. They reported increased BBB disruption, broad vascular damage, and decreased functional performance following middle cerebral artery occlusion (MCAO) [32]. Another rat model that appears promising is the Goto-Kakazaki rat. The rat spontaneously develops Type II diabetes at a young age. Darsalia and colleagues are using the model to look at experimental therapeutics, some of which are used to limit stroke severity [33,34].

A few other factors to consider in the study of diabetes and stroke are genetic predisposition and lifestyle choices. In mice, genetic modification has successfully been used to create a diabetic strain called db/db. Kumari and colleagues used this model to show that diabetes increases the activity of matrix metalloproteinase-9 (MMP-9) following MCAO. MMP-9 causes degradation of tight junctions, disruption of the BBB, and increased neutrophil invasion [35]. A few groups have also looked at how lifestyle factors, such as poor diet and little exercise, increase the severity of diabetes mellitus Type II. Israeli sand rats provide a great model for studying diabetes. Normally the vegetarian rats have few health problems, but if they are fed high-fat Western chow diets, the animals develop hyperinsulinemia, hyperglycemia, cellular resistance to insulin, and obesity [36]. Since the diet can be altered at any time-point, this model provides the flexibility that is often needed in stroke research.

#### 2.2.2. Hypertension Models

Primary hypertension is diagnosed clinically as an elevated systolic and diastolic blood pressure. Sustained levels of hypertension can lead to microvascular damage and changes in protein expression or translation following gene mutation [37]. For example, NOTCH3 is mutated through the selective pressure on small blood vessels occurring as a result of hypertension. As NOTCH3 is associated with the response to mechanical stress and modulating vascular smooth muscle cell phenotype, mutations in NOTCH3 can result in blood vessels undergoing a transformation with medial thickening and luminal narrowing [38,39]. Hypertension also exacerbates atherosclerosis further inducing luminal narrowing. These microscopic changes in the cerebral vasculature caused by hypertension can increase the risk for stroke [40]. Not surprisingly, long-term antihypertensive therapy decreased the risk for stroke in clinical trials [41]. Although primary hypertension is idiopathic, some factors exacerbate its severity. Diets high in NaCl increase the preponderance of hypertension in the population [42]. Genetic altercations in the renin-angiotensin system are linked to increased hypertension [43]. Age itself escalates blood pressure for the majority of individuals [44]. It is therefore necessary to have an animal model that spontaneously develops hypertension with age. It is also important that a Western diet be used to induce changes in blood pressure.

The primary model used to study hypertension and stroke is the spontaneous hypertensive rat stroke prone (SHRSP). The animals have dysfunctional baroreflex sensitivity and therefore develop hypertension and stroke spontaneously as they age [45]. The development of hypertension is accelerated when the animals are fed a high-fat, high-salt diet [46]. This model has been used to confirm white matter lesions and increased levels of MMP-9 following stroke [47]. MMP-9 breaks down the collagenase matrix near cerebral blood vessels following a stroke, which, in conjunction with elevated inflammatory markers, augments BBB disruption [48]. Disruption of the BBB allows for the entrance of inflammatory cytokines and immune-associated cell types that can lead to further damage [48]. Additionally, oxidative stress caused by high blood pressure increases the infarct size of the stroke in this model [49]. The mechanism by which this occurs is through an increase in free radical formation following stroke. The free radicals cause increased neuronal damage by acting on polyunsaturated fats in neurons [50]. Neurons are dense in polyunsaturated fats and the brain contains few antioxidant enzymes capable of eliminating these free radicals making the brain highly susceptible to oxidative damage [50].

#### 2.2.3. Obesity Models

Obesity is increasing at an alarming rate across the globe. Obesity is a known risk factor for diabetes, hypertension, coronary artery disease, and stroke [51]. White adipose tissue produces cytokines such as adiponectin, resistin, and tissue necrosis factor-alpha (TNF-α) [52]. The encoding gene for adiponectin is located in the same area as the genes that produce the metabolic syndrome and vascular disorders [53]. Total adiponectin activity is associated with elevated stroke risk. This occurs due to adiponectin increasing inflammation, insulin resistance, and vascular degradation [54]. Resistin is upregulated with obesity and results in cell uptake of low-density lipoprotein cholesterol, foam cell formation in macrophages, and activation of endothelial cells via upregulation of endothelin-1 and vascular cell adhesion molecule-1 [55]. TNF-α activates both apoptotic and neuroprotective pathways but an increase in apoptotic activity is associated with obesity [56].

The primary model used to study obesity and stroke is the obese Zucker rat. The obese Zucker rat is homozygous for the fa/fa allele, which results in a faulty leptin receptor and hence, the brain fails to respond to satiety signals properly [57]. Following stroke, obese Zucker rats have greater middle cerebral artery remodeling and increased infarct volume in comparison to leaner animals [58]. A proposed mechanism for the increased vascular remodeling is obesity-induced hypertension [59]. A potential cause of greater infarct volume is an elevation of ROS [60]. This model is also useful in studying comorbidities, such as diabetes and hypertension, since they can be induced with the Western diet [61].

The models incorporating diabetes, hypertension, and obesity offer a promising alternative to study stroke. Since the successful implication of tPA in clinical practice, over a 100 drugs for stroke treatment have gone to clinical trial and failed to proceed due to limited efficacy and safety [62]. The therapeutic targets were based on work done in young healthy animal models. Incorporating comorbidities and/or aging into pre-clinical models will allow researchers to isolate pathways that are more likely to be seen in human populations. This will allow for better selection of therapeutic targets.

### 2.3. Accounting for Lifestyle Influences: Effects of Altered Sleep-Wake Patterns

In addition to the incorporation of comorbidities in ischemic stroke models, preclinical studies need to address the effect of variations in lifestyle that may lead to altered risk or outcome. An example of a lifestyle alteration that needs to be addressed is sleep deprivation and/or fragmentation. Sleep plays an integral role in everyday life, consuming approximately one third of a person’s day. Epidemiological studies have shown sleep to be important to a person’s health, with a positive association seen between sleep disruption and morbidity and mortality [63–66]. People who report having short sleep durations, durations of ≤6 h a night, have an increased prevalence of Type II diabetes, hypertension, obesity, cardiovascular disease, and stroke [67–74]. A study in Finland found that people engaged in shift work, defined as work outside regular daytime hours, had a higher incidence of stroke [75]. Sleep disruption is a characteristic of shift work, and people who are engaged in shift work are more likely to have shorter sleep durations than people who work regular daytime hours [76]. According to the 2010 National Health Interview Survey (NHIS), over 40 million employed U.S. adults report having short sleep durations. Therefore, the specific functions and molecular mechanisms of sleep are an active area of research.

In an effort to understand sleep, studies evaluate the consequences when sleep is taken away. In animal models, the function of sleep is analyzed predominantly by preventing the animal from sleeping, termed sleep deprivation (SD). Sleep fragmentation disrupts the quality of sleep without decreasing total sleep time and is less studied. Unfortunately, the method of sleep deprivation is highly variable within the literature and makes results problematic to interpret. Some SD methods place the rodent in a constantly rotating drum or on a platform (“disk-over-water” or DOW) where the animal must continuously move and cannot fall asleep [77,78]. Other methods place the rodent inside an automated running wheel that is activated when the animal falls asleep via electromyogram (EMG) monitoring [79]. The gentle handling method, where the animal is lightly poked or brushed when sleep is observed, is considered to be one of the least stressful methods for the animal [77]. However, this method is not automated and can be taxing on the researcher. Consequently, it is usually only used for acute SD studies of 6 hours or less. Duration of SD is also variable with acute durations of 4 or 6 h, chronic SD of 24–72 h, and repeated bouts of acute SD for days or weeks. This degree of variation makes the ability to interpret the results and generalize between studies problematic.

Within the stroke literature, acute SD prior to cerebral ischemia or injury has been shown to attenuate the severity of the injury, and could possibly be neuroprotective [80,81]. These findings are contradictory to epidemiological studies [74,75]. However, the sleep patterns in the animal studies have normal sleep and then experience shortened sleep duration prior to cerebral insult where the general population experience short sleep durations habitually. Consequently, studies evaluating the effects of sleep disruption on stroke risk and stroke severity need to more closely replicate the sleep patterns seen within the general population. Furthermore, the importance of sleep quality and continuity needs to be further evaluated since fragmented sleep is another characteristic of sleep disruption shown to have negative consequences [82]. Replicating the sleep patterns of the general public in animal studies will allow us to identify the mechanisms and signaling pathways taking place in the human population and discover where intervention is possible to reduce the poor health outcomes associated with sleep disruption.

### 2.4. Identification of Clinically-Relevant Endpoints for Preclinical Studies

In addition to the incorporation of animal models more consistent with the clinical population afflicted by stroke, whether that includes the various comorbidities or other lifestyle influences, preclinical models also likely need to emphasize functional or behavioral measures rather than strict histological analysis. Preclinical studies have long emphasized measurements of infarct volume in assessing therapeutic efficacy despite the fact that the emphasis clinically is on restoration of function and assessment of functional ability. In fact, a clinical study conducted by Saver and colleagues demonstrated that infarct volume serves as a relatively poor surrogate measure of functional outcome with subacute infarct volume displaying only a moderate correlation with 3-month clinical outcome [83]. Consequently, it is clear that preclinical studies may be improved by increasing the emphasis of functional outcome rather than infarct volume, a fact that has been recognized by leading advisory groups in the stroke community such as Stroke Therapy Academic and Industry Roundtable (STAIR) [84]. While multiple groups have performed infarct volume-function correlation studies in preclinical models, these models have employed young-adult animals rather than the potentially more clinically-relevant aged animals or those with comorbidities, limiting the ability to conclude whether volumetric measurements correlate and to what extent with functional capability. Other potentially relevant endpoints of use include the use of diagnostic imaging, such as magnetic resonance imaging (MRI), and cerebral blood flow measurements that are commonly used in the clinic. These may be particularly useful when performing longer duration studies to track infarct evolution over time and also to assess the therapeutic window of opportunity.

## 3. Promising Directions and Potential Targets in Neuroprotectant Development

Despite past shortcomings in neuroprotectant development, multiple pharmacologic agents are currently under investigation in advanced phases of clinical trial development and appear promising based on early phase results. This includes Arundic Acid (ONO-2506), an astrocyte modulator, and minocycline, an inhibitor of microglia.

### 3.1. Modulating Astrocyte Activity

While neuroprotection research has been largely neurocentric in nature and failed to emphasize the prominent role of glia in both homeostasis and injury response, the suggestion has been made that preservation of neuronal survival and function may not be possible without significant regulation of the microenvironment by astrocytes [85]. Astrocytes, frequently described as “housekeeping cells of the nervous system”, are involved in regulating blood-brain barrier (BBB) integrity, removal of metabolic waste products, release of neural growth factors, and uptake of excess neurotransmitter release during synaptic activity [85]. As such, astrocyte modulation represents a promising strategy for future therapeutic development for ischemic stroke [85]. The effect of ischemia on astrocytes is widespread in that signaling amongst astrocytes, as well as between astrocytes and neurons or microglial cells, is likely altered in addition to the potential for necrotic or apoptotic cellular death. This disrupted signaling likely has significant consequences on the control of basic physiologic functions including cerebral blood flow, glutamate uptake by astrocytes, and the ability of astrocytes to serve as an energy reserve during ischemia [86–89].

Perhaps the hallmark event involving astrocytes following neural injury, and specifically ischemia, is the development of reactive gliosis and associated morphologic changes and altered gene expression [11]. One of the challenges in modulating astrocyte activity, and associated gliosis, for therapeutic benefit is the emerging evidence that astrocytes have both protective and detrimental roles, potentially a result of multiple astrocyte subtypes [11]. Recent work by Zamanian, *et al.* have illustrated this concept in that ischemia appears to promote a protective astrocytic response whereas neuroinflammation induced by lipopolysaccharide (LPS) produces a potentially detrimental phenotype. Notably, these two different stimuli each induced significantly altered gene expression yet the expression shared between stimuli was less than 50%, furthering the notion of a stimulus-specific astrocyte response or multiple astrocyte subtypes [11]. The influence of astrocytes on outcome following neurologic injury, and the potential for conflicting roles (beneficial or detrimental), has been seen clearly in a variety of preclinical models including ischemia, experimental autoimmune encephalitis (EAE), and spinal cord injury to name a few. In a preclinical model of ischemia, Li and colleagues demonstrated a protective role of astrocytes by knocking out glial fibrillary acidic protein (GFAP) and vimentin [90]. Deletion of this combination of intermediate filaments, characteristic of astrocytes, resulted in infarct volumes two to three times larger than in wild-type animals [90]. In spinal cord injury, a dual role of astrocyte reactivity has been observed in which initially, astrocytes appear to modulate inflammation and contain inflammatory cells to the damaged region. On the other hand, astrocytes are the primary component of the glial scar that prevents axonal re-growth in the chronic period post-injury [91]. In a model of demyelinating disease, experimental autoimmune encephalitis (EAE), targeted genetic removal of reactive astrocytes resulted in diminished perivascular scar formation and increased leukocyte infiltration into brain parenchyma [92]. The exacerbated inflammatory response was associated with a detrimental outcome [92].

Despite the expanded understanding of astrocyte roles in homeostasis and disease, clear knowledge gaps remain. This includes more clear elucidation of the timing of beneficial versus detrimental responses to injury, how these responses can be pharmacologically manipulated, and how the astrocyte response is altered in association with disease comorbidities and the aging process. Our laboratory and others have begun to explore these factors with the hope of improving the understanding of neural injury in order to develop future therapeutics [93,94].

One potentially promising therapeutic that alters astrocyte activation, and synthesis of S-100B, that has advanced to clinical trials is (*R*)-(–)-2-propyloctanoic acid (ONO-2506). S-100B is expressed by reactive astrocytes throughout the penumbral region following ischemia and leads to nitric oxide release from the astrocyte, potentiating neuronal cell death [95,96]. In an early phase clinical trial, ONO-2506, also known as arundic acid, proved safe and produced a trend towards functional improvement based on the National Institutes of Health Stroke Scale (NIHSS) [95].

### 3.2. Inhibiting Effects of Microglia

Much like astrocytes, microglial modulation may represent a promising therapeutic target for ischemic stroke based on the progression of compounds such as minocycline in clinical trials. Microglial activation, a hallmark of the inflammatory response within the central nervous system post-stroke, has been described as both beneficial and detrimental in nature [97]. Ablation of Mac-2 positive microglia, a marker of activated microglia, resulted in enhanced infarct volume and altered temporality of pro-inflammatory cytokine release. These changes were associated with a nearly three-fold increase in apoptotic cells and a nearly two-fold decrease in IGF-1, a neurotrophic factor, levels [98]. In contrast, numerous other studies have shown an improvement in outcome following ischemia by reducing microglial activation [99,100]. This is achieved by administration of minocycline, traditionally used as an antibiotic, which has been associated with diminished Iba-1 expression, an activation marker, by microglia [99]. Importantly, the microglial response post-stroke begins rapidly but takes hours to days to develop fully, potentially resulting in an extended window of therapeutic opportunity [97]. The idea of an extended window, at least in comparison to that used for thrombolytic therapy, is consistent with preclinical findings in which a clinically relevant dosage of minocycline decreased infarct volume at both 4 and 5 h post-stroke while reducing functional deficits with 4 h administration [101]. In addition to the effects on microglia, minocycline has been shown to alter the function of other immune cells previously implicated in ischemic damage such as macrophages [102]. While not previously investigated specifically with regards to ischemia, the effect of minocycline on macrophages may be related to alterations in macrophage recruitment and/or macrophage polarization, a topic that is discussed at greater length in subsequent sections. The effect of minocycline on subsequent inflammatory processes is logical in that microgliosis following injury is associated with not only the acute and rapid activation of microglial cells but also the recruitment of marrow-derived inflammatory cells that migrate to the brain and penetrate the parenchyma [98]. The precise mechanism via which minocycline mediates protection through microglial-mediated effects remains unclear but has been shown to decrease expression of high-mobility group protein B1 (HMGB1) and *in vitro*, reduces oxidative stress via diminished superoxide release [99,103]. Minocycline may also prove beneficial in other reparative therapies post-stroke, such as stem-cell delivery. Sakata and colleagues demonstrated that minocycline pre-conditioning of neural stem cells prior to transplantation resulted in production of beneficial paracrine factors and diminished graft cell death via upregulation of antioxidant genes associated with Nrf2 [100].

In early phase clinical trials, minocycline has exhibited similar promise in that it has been proven safe when administered alone or in combination with thrombolytics and also exhibited potential efficacy in these small studies. Namely, NIHSS and modified Rankin Scale (mRS) scores were lower, and Barthel Index (BI) scores higher, in the group given minocycline [104]. This improvement was seen as soon as 7 days post-stroke and persisted at day 30 of follow-up [104]. While a large efficacy trial is needed to confirm preliminary findings, minocycline appears promising for the treatment of ischemic stroke [105]. Notably, clinical studies have also shown that minocycline administration results in diminished levels of matrix metalloproteinase-9 (MMP-9) [106]. Preclinical studies have shown a reduction in blood-brain barrier (BBB) permeability following minocycline administration and due to previous associations between BBB permeability and MMP-9 activity, it would appear that these clinical findings indicate a potential effect of minocycline on restoration or maintenance of BBB integrity [103,106].

### 3.3. Modulating the Blood-Brain Barrier (BBB)

As focus has gradually shifted from solely the neuron to include glial cells and the associated neurovascular unit, the role of the blood-brain barrier in ischemic injury has received renewed interest. Much of the work involving BBB permeability has focused on potential mechanisms of degradation, such as matrix metalloproteinases, creating a natural target for pharmacologic agents [107–111]. In work by Pfefferkorn and colleagues, the authors showed the ability to reduce BBB disruption and improve survival following delayed tPA administration by using BB-94, a matrix metalloproteinase inhibitor [112]. In more recent work using a model of traumatic brain injury, a similar phenomenon was shown using Kollidon VA64, a pharmacological agent described as resealing membranes [113]. Improving membrane integrity resulted in diminished neural injury and improved functional outcome [113]. It is clear that more investigation is required to understand not only the role of BBB permeability in disease but also how to target BBB permeability therapeutically, but studies such as those described herein provide a potential proof-of-concept and perhaps most importantly, further indicate the need for a comprehensive approach to ischemic stroke therapeutic development.

## 4. Targeting Inflammation

As demonstrated by the early phase success of agents currently under investigation, such as minocycline and arundic acid, targeting inflammation may offer promise in the quest for identification of neuroprotective compounds. Inflammation offers many opportunities and targets for modulation, many of which persist for hours to days, sometimes even longer, following injury. Consequently, these targeted therapeutics are more likely to be administered within the window of opportunity for a given pathologic process compared to some of the previously tested agents that target events occurring almost immediately post-stroke. Inflammation, while generally thought of in a negative connotation, is an essential component of recovery and repair-associated processes as well, further complicating experimental investigations. In this work, we focus on the dual responses associated with inflammation, particularly with regards to macrophage polarization, an emerging topic in both the inflammation and neuroscience literature.

### 4.1. Inflammation: A Deleterious Event or a Beneficial Response?

The inflammatory process is further complicated due to the fact that various elements can serve a deleterious or a beneficial role in the injury and recovery process, as highlighted earlier in this work with discussion of astrocytes and microglia. Another prime example of this potentially dual response to neural injury is that of macrophages. Macrophages and microglia may contribute to secondary injury processes via production of inflammatory cytokines and reactive oxygen species while also potentially reducing injury through maintenance of the cellular microenvironment by phagocytic clearance of cellular debris and apoptotic cells [114–116]. Emerging evidence indicates that many of these inflammatory cell types may never truly exist in a resting state and rather may almost constantly be undergoing changes in state through intermediary forms, evident by both morphologic and functional changes, in response to the cellular environment and signals from the surrounding regions [115]. This is perhaps most adequately described, and potentially targeted therapeutically, by macrophages. In the following section, we attempt to highlight some of the recent developments in the macrophage literature, particularly those related to various macrophage subsets and neural injury.

### 4.2. Introduction to Macrophage Polarization

Macrophages have long been recognized as a key element in the inflammatory response, particularly in reference to invasion of the body by foreign material such as bacteria. Besides their role as immune effector cells, macrophages are perhaps most important for normal homeostatic processes such as cellular turnover and clearance of cellular debris, hence the description of macrophages as the “janitorial” cell of the body [117]. For example, macrophages are intimately involved in the clearance of over 200 billion erythrocytes each day as well as clearance of apoptotic cells [117]. Consequently, macrophages respond to both endogenous stimuli produced through injury as well as to foreign cell types [117]. As such, work in both the nervous system as well as other body systems indicates that macrophages can be shifted to either a pro-inflammatory or anti-inflammatory state depending on disease state and environmental cues [114]. Gaining a better understanding of the processes mediating this phenotypic shift may lead to therapeutic advances [114]. Perhaps the greatest challenge in this process is the complexity of elucidating these mechanisms *in vivo. In vitro* studies allow for rapid stimulation of inflammatory cells with antigens or cytokines but this response often does not translate to *in vivo* work, or is at the very least, often modified in a significant way [118].

### 4.3. M1 *versus* M2: Classical *versus* Alternative Activation

**Figure 1:**
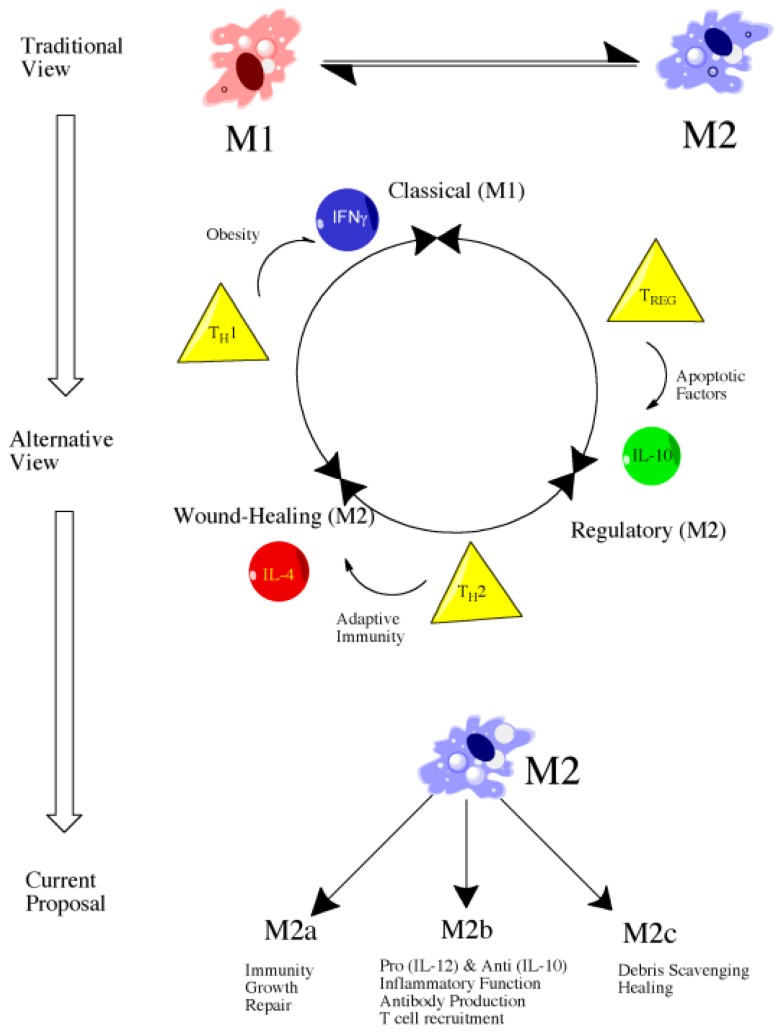
Evolution of views of macrophage polarization from the more simplistic M1 or M2 characterization without subtypes to the alternative or developing view with the M2 population divided into “wound-healing” and “regulatory” subtypes. Even more current proposals identify three populations of M2-related macrophages involved in a range of functions.

Literature focusing on macrophage polarization and subtypes has followed a similar path to that of T-cell literature and, consequently, macrophages have largely been classified as M1 or M2, a description that persists despite evidence showing greater complexity, particularly amongst the M2 population (Figure 1) [117]. In spite of the potential for additional subtypes, we use the conventional M1 and M2 descriptions for organizational purpose but do explore the potential for various M2 subtypes within this work.

### 4.4. Classically Activated Macrophages (M1)

Classical macrophage activation refers to those designated as effector cells as part of cell-mediated immune responses. In the past, classical activation occurred in response to two primary signals—interferon gamma (IFNγ) and tumor necrosis factor (TNF) [117]. This results in increased secretion of pro-inflammatory cytokines and mediators such as IL-12, IL-23, IL-1β, TNF-α, reactive oxygen species (ROS), and nitrosylated species (NS), ultimately producing increased microbicidal or tumoricidal capability [114]. While emerging evidence indicates that other signals may also induce an M1 response, such as certain toll-like receptor (TLR) agonists, the idea remains the same in that M1 macrophages, while vital for host defense, must be carefully regulated due to the possibility of host-tissue damage with unchecked activation [117]. Persistent inflammation and the associated host-tissue damage is a hallmark of numerous autoimmune diseases such as rheumatoid arthritis and inflammatory bowel disease, demonstrating the need for regulation and strict control of activation processes [117].

### 4.5. Alternatively Activated Macrophages (M2)

Alternative macrophage activation, similar to classical macrophage activation, can occur in response to both innate and adaptive signals [117]. The primary signaling molecule responsible for activation is interleukin-4 (IL-4), a cytokine released upon initial tissue injury as well as upon initiation of the adaptive immune response. Alternatively activated macrophages are largely described as beneficial, particularly when it comes to wound healing. Specifically, alternative macrophage activation is responsible for production of extracellular matrix, a property essential in the reparative response. Alternatively activated macrophages are also associated with a reduced expression of pro-inflammatory cytokines, namely TNFα, IL-1β, IL-2, IL-8, IL-12, and CXCL10 [114]. The response of IL-4 stimulated alternatively activated macrophages is much more characteristic of a T_H_2-mediated response in comparison to classical macrophage activation which more closely parallels that of a T_H_1 response [114]. Despite the previous characterization of alternatively activated macrophages as a distinct cellular population, emerging evidence indicates the presence of further subtypes under the M2 umbrella and that they are involved in a variety of functions ranging from wound-healing to regulatory functions. In fact, some have described M2 macrophages as actually three distinct subtypes—M2a, M2b, and M2c, each of which is generated through a unique polarization process and performs distinct functions (Figure 1) [114]. M2a and M2c have been attributed clear anti-inflammatory and reparative roles whereas M2b are somewhat unique in that they produce high amounts of IL-10, an anti-inflammatory cytokine, but low-levels of IL-12, a pro-inflammatory cytokine [114]. Similarly, M2b macrophages may produce TNFα, IL-1β, and IL-6, indicating a more complex role in the inflammatory response than previously recognized [114].

### 4.6. Future Directions: Therapeutic Targeting of Macrophage Subsets

Therapeutically, it is clear that shifting from the pro-inflammatory macrophage phenotype, M1, to the anti-inflammatory phenotype, M2, may confer an advantage in both initial injury and long-term recovery. How precisely to alter the phenotype and at what time post-injury is not clear but the idea is promising, particularly considering that recent studies have demonstrated that macrophage subsets may not be truly distinct entities and rather may undergo switching. This idea has been demonstrated *in vitro* in which activating macrophages with LPS resulted in diminished reactivity to subsequent pro-inflammatory stimuli [119]. In contrast, these macrophages maintained the ability to express other genes associated with anti-inflammatory activities such as IL-10 [119]. The ability to diminish responsiveness to pro-inflammatory stimuli while maintaining the anti-inflammatory capabilities is referred to as endotoxic tolerance and is generally associated with a switch from the M1 to M2 phenotype [119].

While inflammation is clearly a promising therapeutic target, our understanding of inflammatory processes and when precisely each event occurs remains somewhat unclear and likely varies across individuals. As such, a need exists for not only improved measurement and insight into inflammatory processes but also a personalized approach, a common theme discussed throughout medical research in recent years. One method for potentially achieving both these goals is the use of blood-based or CSF-based biomarkers.

## 5. Gaining Insight from the Bedside: Utility of Biomarkers

### 5.1. Biomarkers for Diagnosis and Prognosis

**Figure 2:**
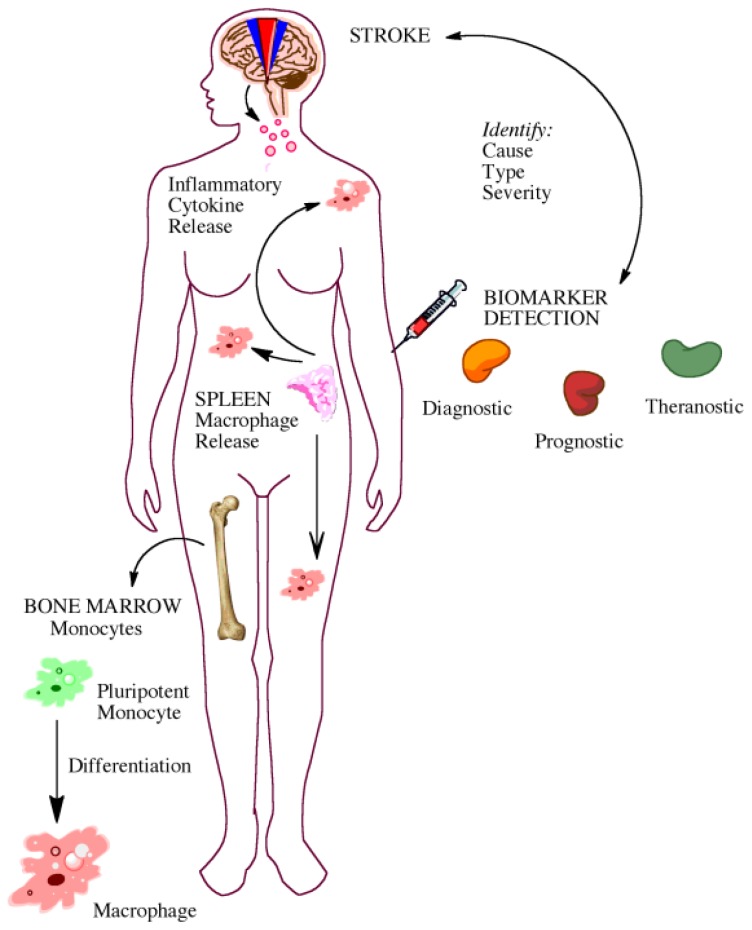
Potential uses of biomarkers include identification of stroke cause, type, and severity allowing for potential diagnosis, prognostication, and therapeutic selection or monitoring (theranosis). Biomarkers may also play a role in understanding disease pathophysiology, particularly in regards to inflammation and macrophage release and/or polarization.

With the recent advances in technology and research, many look to biomarkers as a new avenue to further understand, diagnose, and treat diseases. A biomarker is a biological component that can be tangibly quantified to understand normal or pathological functioning in the body (Figure 2) [120,121]. The roles of biomarkers in the laboratory and clinical setting are vast. They can be used in diagnosing pathologies, monitoring progress of disease or injury, identifying targets for intervention, and assessing risk of pathology.

The measurement of biomarkers translated successfully from bench-to-bedside with troponin-1, a well-known example of a biomarker used to aid in diagnosis of myocardial infarction [122]. Within the neuroscience community, biomarker research has become an area of increased interest and investigation for a variety of pathologies ranging from stroke to traumatic brain injury (TBI) to Alzheimer’s disease (AD). The remainder of our discussion of biomarkers will focus on ischemic stroke, but it is important to recognize the similarities of many disease states, particularly stroke and TBI.

Timing of an accurate diagnosis is essential for patients with stroke. The only FDA-approved treatment, tissue plasminogen activator (tPA), has traditionally had a 3-h window for administration after onset of stroke [123]. While recent guidelines suggest up to a 4.5 h window [124], tPA administration has the potential to result in numerous well-documented adverse effects, such as hemorrhagic transformation (HT) or even death [125]. Consequently, numerous contraindications for tPA administration exist and many patients go untreated. The identification of biomarkers for stroke therefore may not to be limited to traditional diagnostic or prognostic avenues but also more appropriate identification of patients at risk for tPA-associated morbidities. Likewise, biomarkers may also be useful in determining patients in which tPA administration may be beneficial despite the presence of a comorbidity or risk-factor that would normally result in exclusion from tPA-based treatment.

Previous studies have demonstrated differences in numerous markers that may one day potentially play a role in detection of ischemic stroke, stroke-mimic, and transient attack [126,127], as well as predict patient outcome [126–128], determine stroke severity, predict the risk of recurrent stroke [128] or hemorrhagic transformation (HT) [129], increase understanding of pathophysiology behind cerebral ischemia [130], and provide possible targets for therapeutic intervention [131]. While additional studies are certainly required to elucidate the most appropriate use of the markers discussed in the cited studies, each of these studies has provided valuable insight and potential examples of biomarker utilization at various time-points in the therapeutic or prognostic decision-making process, particularly as related to patient suitability for tPA administration.

Historically, biomarker-based studies sought the identification of a single marker for diagnostic/prognostic purposes. However, the investigated biomarkers have proven to be not specific or sensitive enough across multiple populations for clinical usage [120]. Thus researchers have transitioned largely towards identification of a panel of biomarkers rather than a single marker. The discovery of a group of biomarkers, instead of a sole marker, may improve the specificity, sensitivity, and prediction values across time [120,129]. A panel could therefore additionally provide the healthcare provider with more information than a single biomarker could reliably do alone [129]. Due to biomarker discovery and investigation being a relatively new field, our review and discussion below is limited primarily to single marker studies.

Multiple biomarkers of stroke have been identified in the literature. A popular biomarker of note is matrix metalloproteinase 9 (MMP-9). High levels are correlated with current neurological deterioration, stroke severity, and infarct size [132]. Interestingly, before administration of tPA, high levels also have been shown to accurately predict HT [133,134]. Despite MMP-9 playing a clear role in stroke pathology, a major pitfall of its use in the clinical setting is that it is not specific to the brain. It is involved with inflammation and matrix remodeling throughout the body and in different pathologies such as diabetes [135], atherosclerosis [136], hypertension [135] and many others. Therefore, MMP-9 likely cannot be used as a stand-alone biomarker for stroke; however it may be a helpful addition to a biomarker panel.

Another biomarker of note is glial fibrillary acidic protein (GFAP). High levels of GFAP have been found to be indicative of hemorrhagic stroke in the acute stage, and therefore, GFAP may be an important biomarker to consider when deciding the appropriateness of thrombolytic administration [137]. Importantly unlike MMP-9, GFAP is released strictly by astrocytes in the brain [138]. Outside of stroke, GFAP possesses prognostic value for outcome in cases of mild and severe traumatic brain injuries, though it is suggested that it is not an independent factor [139,140].

A high concentration of S100 calcium binding protein (S100B) also has the ability to distinguish between hemorrhagic and ischemic stroke [141] and positively correlates with infarct volume [142]. When measured along with MMP-9, Glickman and colleagues revealed that S100B levels can differentiate between ischemic stroke and stroke mimic [143]. Yet also like MMP-9, evidence indicates that S100B is expressed outside of the brain, such as in cancer, and therefore suffers from the same lack of specificity associated with other markers previously mentioned [144,145].

D-dimer concentration shows promise as a biomarker of stroke. As a component of a blood clot, d-dimer is useful for diagnosing the subtype [146], specifically cardioembolic stroke [147], and distinguishing between ischemic stroke and stroke-mimic [125]. As a thrombotic biomarker, d-dimer has many uses in regards to stroke, but it too is found throughout the body and, therefore, is not reflective solely of central nervous system events. Regardless, d-dimer has proven useful in the diagnosis of post-stroke cardiovascular complications such as pulmonary embolism, deep vein thrombosis and others [143].

The idea of individualized therapy, particularly when it concerns the selection of patients for thrombolytic therapy, has been further explored in recent years by Ning and colleagues. They showed significant prolonged effects on cellular signaling following tPA as evident by prolonged patterns of degradomic protein expression up to 5 days post-treatment, a finding in distinct contrast to that of non-tPA treated stroke patients in which a hyperacute response was evident but ceased by 24 h [148]. Besides demonstrating widespread effects of tPA on the extracellular matrix and brain parenchyma, far outside of the acute therapeutic window, their work could potentially demonstrate the utility of biomarkers in identification of patients at risk for adverse effects following therapeutic administration.

### 5.2. Biomarkers for Elucidating Disease Pathophysiology

While biomarkers are often thought of primarily for diagnostic and prognostic purposes, emerging work indicates a potentially greater role for biomarkers in elucidation of disease pathophysiology, especially communication between the brain and systemic responses. For example, Hayakawa and colleagues showed that by disrupting either the release of high-mobility-group-box-1 (HMGB1) from reactive astrocytes, or the HMGB1 receptor on endothelial progenitor cells, neurovascular remodeling is altered, and worsened deficits result [149]. This work demonstrates the ability to isolate cell-specific events by taking *in vitro* based findings, such as the release of HMGB1 into astrocyte-conditioned media, and applying them to *in vivo* studies to determine the effect of HMGB1 systemically on post-ischemic injury. Other studies have used *in vitro* models in a similar fashion to demonstrate complex signaling relationships amongst neurons and glia. Specifically, Chu *et al.* showed the protective role of factors released by ischemic microglia, astrocytes, and neurons on astrocytes subjected to ischemia [150]. While space constraints prevent the mention of all applicable works, studies such as those described illustrate the potential for an expanded role of biomarkers in elucidation of disease pathophysiology and crosstalk between various cells within the brain and systemically.

## 6. Discussion

The lack of successful therapeutic translation from bench-to-bedside for a devastating disease such as stroke, despite the progression of over 100 proposed therapeutics to clinical trials, necessitates a closer review of both how stroke research is conducted as well as the therapeutic targets selected. While preclinical studies have improved in rigor, particularly with the advent of the Stroke Therapy Academic and Industry Roundtable (STAIR), clear deficiencies remain. Perhaps most notably, the lack of emphasis on the aging process and commonly associated comorbidities found in stroke patients, is persistent throughout the literature. As aging is the greatest risk factor for stroke, and numerous works have identified a role of aging in response to injury, inclusion of more preclinical models utilizing aged animals may lead to development of promising therapeutics. Other promising avenues of investigation in stroke research include the role of glia, particularly with regards to the inflammatory response. Promising preclinical studies and early phase clinical studies, such as the work with minocycline, indicate a potential protective effect associated with microglial inhibition. Similarly, astrocytic modulation via administration of arundic acid may result in improved outcome. Other areas of investigation with regards to inflammation, such as understanding how to manipulate macrophage polarization for therapeutic benefit may lead to breakthroughs in drug development. Lastly, biomarker discovery, while often thought of in a diagnostic or prognostic sense, may in fact serve a much broader role in understanding disease pathophysiology and therapeutic selection. By improving preclinical models to more accurately emulate the clinical scenario and targeting disease pathophysiology beyond the nearly immediate events, the likelihood of successful translation from laboratory to clinic may be improved.
